# Children's Vulnerability to Sexual Violence During COVID-19 in Kenya: Recommendations for the Future

**DOI:** 10.3389/fgwh.2021.630901

**Published:** 2021-02-24

**Authors:** Laura M. Stevens, James C. Rockey, Sarah R. Rockowitz, Wangu Kanja, Melissa F. Colloff, Heather D. Flowe

**Affiliations:** ^1^School of Psychology, University of Birmingham, Birmingham, United Kingdom; ^2^Wangu Kanja Foundation, Nairobi, Kenya

**Keywords:** sexual violence against children, COVID-19, situational crime prevention, public health approach, offender versatility, Kenya

## Abstract

This article discusses the latest research that reveals that children seem to be facing new risks of sexual violence in Kenya during the COVID-19 pandemic. The evidence suggests there have been changes in patterns of sexual offenses against children coincident with lockdowns, curfews, and school closures. In particular, emerging evidence from Kenya suggests that child victims are younger, more likely to be victimized by a neighbor in a private residence, and in the daytime, compared to pre-pandemic. We conclude that situational crime prevention strategies that focus on providing alternative safe venues to reduce offending opportunities must be a central part of a public health approach to reduce children's vulnerability during crises such as COVID-19.

## Introduction

During the COVID-19 pandemic, many countries have implemented emergency measures to reduce the spread of the disease. However, these measures may be intensifying sexual and gender-based violence [SGBV; (1)]. UN Women (2) found that in the last 12 months, 243 million women aged 15–49 had been subjected to SGBV by an intimate partner. The UN Population Fund (3) estimates that, after 6 months of emergency measures, there will be 31 million additional SGBV cases worldwide. SGBV is exacerbated during crisis situations because of heightened gender inequality and financial pressures (4), as well as difficulties in accessing relevant medical and legal services (5). COVID-19 has further intensified SGBV due to lockdown measures, effectively trapping victims in their homes with potential abusers [for a review of GBV during COVID-19, see (4)].

Specific concerns have been highlighted for COVID-19 and sexual and reproductive health. For example, the postponement of programmes designed to protect girls from female genital mutilation (FGM) and child marriage due to COVID-19 is estimated to lead to two million more cases of FGM and 13 million more child marriages over the next 10 years (3). This perspective piece focuses on low- and middle-income countries (LMICs), and specifically Kenya. This is because there is political good will to research and respond to SGBV in Kenya following President Kenyatta's call to action in the wake of increasing SGBV in the country (6). There is also mounting empirical evidence about new patterns of violence against children, owing to the data collection infrastructure established by the Survivors of Sexual Violence in Kenya Network (7). In Kenya, a reported 29% of women are married before the age of 18 (8). This is expected to increase during COVID-19, as financial strain leads families to marry their daughters to reap economic benefits in contexts where bride price is practiced (9, 10). Additionally, the Kenyan Democratic and Health Survey (8) estimates that 21% of Kenyan women aged 15–49 years had experienced female genital mutilation (FGM). During COVID-19, school closures and limited police presence have left young girls increasingly exposed to FGM (11). A report from October 2020 found that 2,800 12 year-old girls were paraded through the Kuria village in Kenya to showcase their initiation, despite legislation prohibiting FGM (11).

In this article, we discuss how the latest research reveals that children in LMICs such as Kenya seem to be facing new risks of sexual violence during the pandemic. In particular, while previous research has highlighted the adverse consequences of suspending programmes designed to prevent SGBV, we will highlight that patterns of offending behavior have potentially changed following the implementation of lockdowns, curfews, and school closures. These new risk factors must be considered in order to protect children from sexual violence.

### Child Victims in Low- and- Middle Income Countries During COVID-19

Whilst the impact of COVID-19 on sexual violence and children is a global issue, it is of particular concern in LMICs. For example, South Africa saw a 61.6% increase in child abuse disclosures during COVID-19 in comparison to the previous year, with emotional abuse being the most frequent, followed by physical and sexual abuse (12). Additionally, emerging findings suggest that measures used to control COVID-19 in Kenya have exacerbated sexual violence against children, particularly girls. Kenya had high rates of victimization pre-COVID-19, with the National Violence Against Children Survey (13) finding that 13.5% of girls and 2.4% of boys experience sexual violence by the age of 17. However, this escalated further with the emergency measures introduced to prevent COVID-19 spread, including a nightly dusk-to-dawn curfew, travel restrictions, and school closures (6, 7, 14).

Ongoing evidence collection by the Survivors of Sexual Violence in Kenya Network suggests that COVID-19 has changed patterns of sexual violence against children (7, 15). First, child sexual violence victims are now age 12 on average (7), compared to 16 previously (13). This finding is corroborated by evidence from forensic medical examiners at gender-based violence recovery centers in Kenya who have noted that survivors attending hospitals for SGBV violations during COVID-19 are now younger and mostly below the age of 16 (16).

Second, school closures and reduced parental monitoring have coincided with an increase in offenses perpetrated by individuals known to the survivor. Offenses are more often being perpetrated by neighbors during the pandemic compared to before (42 vs. 16%) (7, 13). There are reported cases wherein neighbors have gained access to children through the ruse of providing educational resources, such as laptops and internet access, during school closures (7, 15). Worryingly, it is increasingly being recommended that neighbors should be utilized during COVID-19 to help identify and protect victims, and to aid in reporting incidences [e.g., (1, 17)]. However, prevention and protection programmes must urgently take heed of emerging evidence that neighbors are frequent perpetrators of violence during the pandemic in Kenya.

Third, the timing and location of sexual violence offenses seem to have changed during the COVID-19 pandemic. Offending during the pandemic in Kenya most often is taking place during the day (76% of all cases) when children would have previously been at school (7). Additionally, most sexual offenses against children during COVID-19 appear to be occurring in private locations (71% of all cases), with most of these offenses occurring in the perpetrator's home (65%), followed by the child's own residence [29%; (7)]. This is in stark contrast to pre-COVID-19, when few offenses against children occurred in private (24.5% of all cases), and few attacks occurred at the perpetrator's home (14.9%) or the child's residence [5.4%; (13)].

## Discussion

School closures are associated with many consequences for children, including a lack of access to educational content with 80% of 18 million children not accessing the radio or online content provided by the Government during this time (18). As a result, Kenya's Education Minister has announced that students will be required to repeat this academic year (19). Additionally, the above findings and other previous research highlights that school closures are related to increased sexual violence against children. Whilst boys and girls are both victims of sexual violence, sexual violence disproportionately affects girls with 13.5% of girls experiencing sexual violence by the age of 17 in comparison to 2.4% of boys (13).

Goulds et al. (20) studied school closures during the Ebola epidemic in Sierra Leone in 2014–15, finding that closures left children at greater risk of rape, and led to 65% increase in teenage pregnancies (21). Furthermore, research has found that the financial strain experienced during pandemics increases the number of young girls being forced into child marriage and high-risk work to provide food for their families. Moreover, there is heightened concern that school closures due to COVID-19 will increase children's vulnerability to violence and abuse (22).

Young girls are also at an increased risk of FGM and child marriages whilst they are not able to access the safety of their school networks (23, 24). Schools may offer sanctuary to young girls escaping child marriages and FGM, providing them with shelter, food, education, and the chance of a better future (25). However, school closures during the pandemic may result in young girls being sent home to families which will exploit them, if no one else in the community is able to protect them adequately from COVID-19 or afford to sponsor them (25). Additionally, financial pressures exacerbated by COVID-19 increase the proportion of child marriages, as families require the bridal price paid for their daughters to support their families (23, 25). Child marriages, like rape, lead to teenage pregnancies, with one area in Kenya reporting that 4,000 teenagers have become pregnant during the pandemic (6). Moreover, it is clear that the school closures implemented to reduce the spread of COVID-19 has had far reaching consequences, which disproportionately affect young women and more protective measures need to be endorsed to reduce this (26).

### Versatility of Sexual Offending

Sexual offender specialization and versatility have been widely studied to understand how to prevent and protect people from violence. Shifting patterns in the “who, what and where” of sexual violence seen during COVID-19 illustrate the *versatility* of sexual offending, which means that offenders adjust their offending behaviors to correspond with the offending opportunities that arise (27). Most sexual offenders, including child molesters, are versatile (28). Many sexual offenders do not have a predilection toward victims of a specific age range, but rather victimize people across a range of ages [(29); see also (30)]. Most concerning is that versatile offenders pose a higher risk for future sexual and violent recidivism than non-versatile offenders (31). Thus, it is important for policy makers and duty bearers to recognize that sexually violent behavior evolves with the circumstances. The COVID-19 pandemic has resulted in more unsupervised children and offenders being close to home, likely providing increased opportunities for offenders to target and violate especially younger children. Therefore, protection strategies need to be reviewed and adapted to changing risks.

### Protecting Children: Situational Crime Prevention

Situational crime prevention strategies can potentially reduce immediate offending opportunities that are arising from school closures (32, 33). Situational crime prevention strategies consider key environmental factors that are associated with offending opportunities, and design prevention measures that restrict opportunities, whilst also increasing the risks of offending and limiting the benefits (33–35). One example of situational crime prevention commonly used to address SGBV is the implementation of safe refuge locations, with these primarily run by community groups (34, 36). These shelters increase the safety of survivors whilst also promoting community participation in their protection (36). The prioritization of refuge centers for SGBV survivors is vital during COVID-19 to prevent survivors being isolated with their abusers, as many perpetrators are family members (1, 7, 16, 37).

In combination with temporary housing for survivors, alternative safe environments for children are required during the day whilst schools are closed. This will limit offenders' opportunities to access children during extreme circumstances such as a pandemic (21). These safe spaces for children should be developed by trusted community groups, as they have advanced knowledge of the local population and their needs (34). Alternative safe environments were used in Sierra Leone during the Ebola pandemic (21). In this context the protective space was used for social support, education on sexual and reproductive health, and vocational training to increase the economic potential of the young girls (21). Programs such as these could be implemented in LMICs during COVID-19 to reduce the sexual exploitation of young girls and prevent school dropouts due to teenage pregnancies [(21); see (7) for further recommendations].

One limitation of the situational crime prevention framework is that by limiting an offender's opportunity to target certain victims, offenders may instead pursue other types of victims [e.g., new age groups or another gender; (35)]. This was found in Sierra Leone, where only younger girls accessed the safe space; thus, offenders began violating older girls instead (21). Moreover, alongside crime prevention methods, long-term holistic societal changes are needed to prevent this displacement, such as by tackling social and economic factors that correlate with crime (35).

Violence against women is a systemic issue. Attitudes towards gender equality license SGBV. Therefore, it is essential a wider public health approach is taken to tackle SGBV (38, 39). A public health approach aims to create an environment where all citizens are safe and healthy (38). Therefore, a public health approach would work to adapt a culture of gender equality, where the human rights of individuals were respected to promote safety. This can be achieved alongside a situational crime prevention framework by the implementation of wider community programs, such as education programmes on gender equality, healthy relationship formation, and bystander intervention, as well as economic strengthening programs, such as cash transfers and micro-finance training (40). These programs would work to implement a culture of zero tolerance toward violence, whereby all are equal and financially viable.

However, one weakness regarding research into crime prevention methods is that there is limited empirical data from low-resource environments. It is imperative to increase the capacity of countries like Kenya and South Africa to document cases and research patterns of violence over time (41). This information can be used to monitor and evaluate crime prevention techniques and suggest how they can be improved in the future.

## Conclusion

Emerging evidence from Kenya suggests that COVID-19 and the associated curfews and school closures have coincided with children being violated at a younger age, and increasingly in private residences by individuals known to them, namely neighbors. It is critical that crisis management plans for COVID-19 are altered to explicitly provide for the protection of children, such as by providing alternative safe environments during school closures and increasing the provision of refuge centers. Continued surveillance of patterns of sexual violence against children is vital to identify new risk factors and protect children during COVID-19. Whilst the data presented here are specific to the Kenyan context, these patterns and recommendations should be applied to protecting children globally, especially in other LMIC contexts.

## Data Availability Statement

The original contributions presented in the study are included in the article/supplementary material, further inquiries can be directed to the corresponding author/s.

## Author Contributions

LS, HF, and JR drafted the manuscript, which was reviewed by SR, MC, and WK. All authors contributed to the editing of the approved final manuscript.

## Conflict of Interest

The authors declare that the research was conducted in the absence of any commercial or financial relationships that could be construed as a potential conflict of interest.
